# Coexistence of Pernicious Anemia and Myasthenia Gravis Presenting As Dyspnea

**DOI:** 10.7759/cureus.15295

**Published:** 2021-05-28

**Authors:** Sara Khademolhosseini, Elspeth Springsted, Seyedmohammad Pourshahid, Badri Giri

**Affiliations:** 1 Internal Medicine, Icahn School of Medicine at Mount Sinai, Queens Hospital Center, New York, USA; 2 Internal Medicine, Virginia Tech Carilion School of Medicine, Roanoke, USA; 3 Pulmonary and Critical Care, Virginia Tech Carilion School of Medicine, Roanoke, USA

**Keywords:** myasthenia gravis (mg), pernicious-anemia, dyspnea, multiple autoimmune diseases, coexistence

## Abstract

Dyspnea is a common symptom and may be due to a multitude of conditions, including cardiopulmonary insufficiency, anemia, neuromuscular disorders, obesity, or deconditioning. It is not uncommon that more than one process contributes to shortness of breath. Here, we present a patient with a complaint of worsening shortness of breath who was found to have two very rare causes of dyspnea simultaneously. The symptoms resolved with treatment of pernicious anemia and myasthenia gravis (MG). The coexistence of pernicious anemia and MG is extremely rare, with only two other cases reported so far.

## Introduction

Dyspnea, or subjective shortness of breath, is a common presenting symptom that affects millions of patients. With a wide range of differential diagnoses, dyspnea could be an early manifestation of pulmonary diseases, cardiovascular insufficiency, anemia, neuromuscular disorders, obesity, or deconditioning [1]. It is not uncommon that more than one process contributes to dyspnea. The providers must keep a broad differential diagnosis and investigate additional causes if the patient is not responding to treatment as expected. Although cardiopulmonary-associated shortness of breath constitutes a great proportion of cases, alternative etiologies need to be considered.

## Case presentation

A 73-year-old female with no significant medical history presented to the pulmonary clinic for dyspnea on exertion, gradually worsening over one year. She endorsed generalized weakness and frequent falls. She denied cough, fever, night sweats, or weight loss. On physical examination, she was afebrile with a heart rate of 81 beats/minute, blood pressure 141/86 mmHg, respiratory rate of 16 breaths/minute. Oxygen saturation was 99% on room air. Lung sounds were clear to auscultation. Mild bilateral upper extremity weakness and ataxic gait were noted. Reflexes were brisk in the upper and lower extremities, and the Romberg test was positive.

An extensive cardiac workup was nondiagnostic before referral to the pulmonary clinic, including echocardiography with preserved ejection fraction and normal stress test.

**Figure 1:**
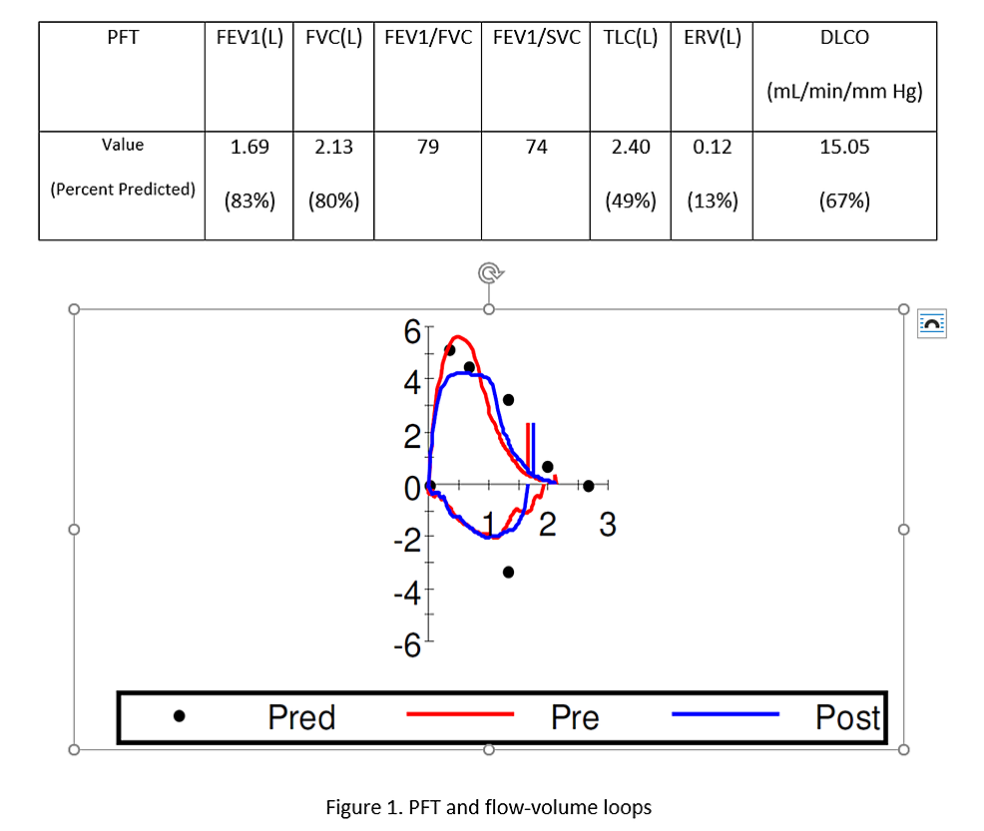
Pulmonary function test and flow-volume loops.

Pulmonary function test (PFT) (Figure 1) revealed a severe restrictive pattern and mildly reduced diffusing capacity of the lungs for carbon monoxide (DLCO).

CT scan of the chest showed no evidence of parenchymal lung disease. The workup for connective tissue disease and hypersensitivity pneumonitis was unremarkable. The remarkable laboratory results are summarized in Table 1.

**Table 1:** Remarkable laboratory results.

Remarkable lab	Value, unit	Interpretation
Hemoglobin	10.3 g/dl	Decreased
Mean Corpuscular Volume	112 fL	Elevated
B12	<150 pg/ml	Undetectable
Folate	11.31 ng/ml	Normal
Homocysteine	102.7 umol/L	Elevated
Methylmalonic Acid	6450 nmol/L	Elevated
Intrinsic Factor Block Ab	Positive	Abnormal
Acetylcholine Receptor Block Ab	27% inhibition	Increased inhibition
Acetylcholine Receptor Bind Ab	2.31 nmol/L	Elevated
Acetylcholine Receptor Mod Ab	76% inhibition	Increased inhibition

Serology was positive for myasthenia gravis (MG). Electromyography and nerve conduction velocity showed a decrement in repetitive stimulation, but it was not detected until 10 minutes post-exercise. The patient was started on pyridostigmine, azathioprine, and prednisone to manage seropositive MG. Her MG medication doses were increased over three months, but her symptoms, especially dyspnea and generalized weakness, did not improve. Intravenous immunoglobulin therapy was started, and fortunately patient had a dramatic response with the first session. The patient reported relief from shortness of breath and weakness. She is currently co-managed by the neurology and pulmonology clinic.

## Discussion

The PFT revealed a severely reduced total lung capacity (TLC), and mild reduction of DLCO and residual volume (RV) (DLCO was within normal limits when adjusted for alveolar ventilation). Forced expiratory volume (FEV1), forced vital capacity (FVC), and their ratio were normal. This pattern suggests a restrictive pattern with a wide range of differential diagnoses categorized into intrinsic lung diseases, the extrinsic disorders of the chest wall and pleura, and neuromuscular diseases [2]. There was no evidence of inherent lung diseases like lung parenchymal pathology or air space disease in chest CT that correlates with this degree of symptoms.

Usually, morbid obesity with BMI above 40 and neuromuscular diseases share some PFT changes such as reduced FEV1, FVC, expiratory reserve volume (ERV), and TLC, unchanged FEV1/FVC, and increased RV. Maximum inspiratory and expiratory pressure may be lower than the expected lower limit of normal for age and sex in patients with neuromuscular disorders [3]. In this case, BMI was only 31, and her extent of dyspnea did not correlate with obesity or deconditioning as she was getting short of breath while talking. No gross chest wall disease or pleural disorder was noted in the investigation. 

Megaloblastic anemia could be contributing to her shortness of breath; however, her hemoglobin was stable and not low enough to explain her severe dyspnea.

B12 deficiency presents a wide range of features like macrocytic anemia, gastrointestinal symptoms, and a variable neurologic abnormality. Symmetric paresthesia and ataxic gait are the most common neurological findings [4]. Mood disorder, forgetfulness, and dementia are reported as well. Muscular weakness that may progress to paraplegia is seen with severe deficiency but is not a common finding [5].

Symptoms of tissue hypoxia may occur with severe anemia or the presence of underlying heart disease. Typically, anemia develops gradually, and the physiologic compensation mitigates symptoms of anemia like shortness of breath and palpitation [4]. Hence, dyspnea is rarely the presenting symptom of B12 deficiency, which could cause shortness of breath by a combination of symptomatic anemia and muscular weakness [4,6,7]. Pernicious anemia is a subtype of B12 deficiency caused by autoantibodies targeting intrinsic factors, interfering with the absorption of B12. Pernicious anemia is associated with other autoimmune conditions such as thyroid disease or vitiligo [7].

At this stage, alternative causes of dyspnea, such as neuromuscular disease was coming higher in the list of differentials. MG is an autoimmune disease that affects neuromuscular transmission. MG usually presents a combination of weakness in ocular, bulbar, skeletal, and respiratory muscles [8]. An anti-acetylcholine antibody has a specificity close to 99%, which makes a false positive extremely unlikely. Also, the sensitivity of serology is 85% in generalized and close to 50% in ocular MG [9]. MG crisis, which causes respiratory failure, usually happens in the late stages of the disease, and patients have prior extensive neurological signs and symptoms. Isolated respiratory muscle weakness caused by MG is very rare and makes the diagnosis challenging [10]. MG is associated with other autoimmune diseases such as autoimmune thyroid disease or connective tissue disorders such as rheumatoid arthritis, lupus, and Sjogren's syndrome [11].

The coexistence of pernicious anemia and MG is extremely rare, and to the best of our knowledge, only two other cases have been reported so far [12,13].

## Conclusions

Dyspnea is a widespread symptom with a broad differential diagnosis, including pulmonary diseases, cardiovascular insufficiency, anemia, neuromuscular disorders, obesity, or deconditioning. Providers must recognize the possibility of contribution from different etiologies. Cardiopulmonary-related dyspnea is one of the most prevalent, but other differentials like anemia and neuromuscular diseases need to be considered as well. In this case, the diagnosis of concurrent pernicious anemia and MG, which are rare causes of dyspnea, led to therapy with resolution of shortness of breath. The coexistence of pernicious anemia and MG is extremely rare, and to the best of our knowledge, only two other cases have been reported so far.
